# Effects of COVID-19 pandemic on structural brain development in early adolescence

**DOI:** 10.1038/s41598-023-32754-7

**Published:** 2023-04-05

**Authors:** L. van Drunen, Y. J. Toenders, L. M. Wierenga, E. A. Crone

**Affiliations:** 1 Leiden Consortium of Individual Development (L-CID), 2333 AK Leiden, The Netherlands; 2 Social and Behavioral Sciences, Erasmus University Rotterdam, 3062 PA Rotterdam, The Netherlands; 3 Leiden Institute for Brain and Cognition (LIBC), 2333 AK Leiden, The Netherlands; 4 Institute of Psychology, Leiden University, 2333 AK Leiden, The Netherlands; 5 Brain and Development Research Center, Leiden University, Wassenaarseweg 52, 2333 AK Leiden, The Netherlands

**Keywords:** Cognitive neuroscience, Social neuroscience

## Abstract

The COVID-19 pandemic caused a global health crisis with large behavioral effects and serious stress and social consequences. Particularly, teenagers suffered pandemic-related social restrictions including school closures. This study examined whether and how structural brain development was influenced by the COVID-19 pandemic and whether pandemic length was associated with accumulating or resilience effects of brain development. We investigated structural changes in social brain regions (medial prefrontal cortex: mPFC; temporoparietal junction: TPJ) as well as the stress-related hippocampus and amygdala, using a longitudinal design of 2 MRI waves. We selected two age-matched subgroups (9–13 years old), one was tested *before* (n = 114) and the other *during* (peri-pandemic group, n = 204) the COVID-19 pandemic. Results indicated that teenagers in the *peri*-pandemic group showed accelerated development in the mPFC and hippocampus compared to the *before-*pandemic group. Furthermore, TPJ growth showed immediate effects followed by possibly subsequent recovery effects that returned to a typical developmental pattern. No effects were observed for the amygdala. The findings of this region-of-interest study suggest that experiencing the COVID-19 pandemic measures had accelerating effects on hippocampus and mPFC development but the TPJ showed resilience to negative effects. Follow-up MRI assessments are needed to test acceleration and recovery effects over longer periods.

## Introduction

The recent COVID-19 pandemic was one of the largest global environmental interventions since decades. This pandemic resulted in a health crisis with large effects on behavior and with several serious social consequences, such as social distancing, social isolation following infection, limited interactions with friends and school closings^1,2^. Even though these behavioral interventions had effects on all individuals, especially teenagers suffered from lower mental-well-being and negative feelings relative to older age cohorts^3,4^. The human brain develops during the teenage years and this process is thought to be shaped and affected by social experiences^5–7^. The teenage years are a time during which young people have the fundamental need to explore, interact with friends and to find their way in larger social circles^8,9^. Therefore, the question arises whether and how COVID-19 related behavioral interventions affected social brain development of teenage children.

Several decades of research has demonstrated that the human brain goes through periods of enhanced growth in development. Researchers have distinguished between grey matter cortical thickness and grey matter surface area as important indices of grey matter brain structure, each showing differential growth patterns. Early in development, there is an increase in cortical thickness between birth and infancy which reaches a relative plateau in childhood, followed by a subsequent decrease in cortical thickness in adolescence. Cortical surface area starts to decrease more slowly in childhood^10–13^. This decrease in cortical grey matter thickness and surface area is explained as reflecting a period of increased efficiency for acquisition of high brain functions in humans^6,14–16^, and longitudinal correlation studies showed that individual differences in development are related to individual differences in experience^17–21^. Moreover, there are marked regional differences in these developmental patterns with the most protracted development within the prefrontal cortex and association cortices^6,12^, including the medial prefrontal cortex (mPFC), temporal parietal junction (TPJ) and superior temporal sulcus (STS), also referred to as the social brain network^7^. Based on twin studies, which allow for the estimation of heritability indices by comparing same-sex monozygotic (sharing 100% of the genes) with dizygotic (sharing on average 50% of the genes) twin pairs, it was previously observed that these social brain regions are affected by both genetic and environmental factors^22^. While cortical thickness and surface area are both highly heritable, a twin-study showed that they are affected by distinct genetic influences^23^. The environmental factors could for example include socio-economic status (SES), as prior studies showed growing up in a low SES environment is linked to accelerated brain development^19,20,24^. Another example of an experience-driven effect was shown in a longitudinal study that showed correlations between grey matter thickness development in the social brain regions and social experiences such as friendship quality, with higher friendship quality being associated with accelerated grey matter development^21^. To date, it has not yet been investigated how the development of social brain regions were affected by the experiences of teenage children in the COVID-19 pandemic, but it is expected that especially the social brain regions, which have the most protracted development during the teenage years and are thought to be influenced by social experiences in the teenage years^6,7,25^, may be affected by the experience of growing up during the COVID-19 pandemic.

In addition to the cortical brain regions, the subcortical brain regions also show developmental changes during the teenage years, but the pattern is less consistent^26–28^. Some studies have reported increases in amygdala volume, a brain region that is involved in the evaluation of the emotional significance of stimuli and thought to be influenced by stressful experiences^29–31^. Previous studies reported a developmental pattern showing that amygdala volume increases by approximately 7% from childhood to mid-adolescence with stable growth after 14 years of age, however, there are significant individual differences^7,32^. Prior work showed that children growing up in exposure to stressful events (e.g., SES disadvantage) showed accelerated growth of volumetric amygdala compared to children of the same age^29,33,34^. A second brain region that is thought to be influenced by stressful and new experiences due to COVID-related pandemic restrictions is the hippocampus^29,30,35^. This brain area is involved in memory and socio-emotional functioning which is closely linked to the hypothalamic pituitary adrenocortical (HPA) axis, a mediator of stressful events^36^. Prior studies indicated that typical development of hippocampus volume shows increases until early adulthood^13,26,28,32,37^. Additionally, other work focusing on effects of stress (e.g., childhood maltreatment) showed that reduced volumetric hippocampus growth was mainly found in adults whereas children showed typical growth, suggesting atypical development after a prolonged period of stress^30,38^. One recent cross-sectional study in 16-year-olds showed that the effects of the COVID-19 pandemic were related to larger hippocampal and amygdala volume by comparing adolescents that were assessed *before*-pandemic to adolescents assessed *peri*-pandemic^39^. Because of the influences of stressful experiences^31,39,40^, we expected that amygdala and hippocampal development would be affected by social isolation during the COVID-19 pandemic and we investigated this question in the present study using a longitudinal design, which allowed us to test whether brain development is affected by measures related to the COVID-19 pandemic.

Finally, we also assess whether there is an association between the duration (i.e., length) of the pandemic effect on brain development. Some studies suggest that immediate social influences have a large effect on brain development but that individuals recover by showing resilience to negative effects^41–43^. Other studies suggest that longer exposure to negative events can have accumulating effects on brain development^19,20,24^. Evidence for resilience effects during the COVID-19 pandemic have so far only been observed for behavioral measures. Empathy and prosocial behavior are cornerstones of social behavior such as in friendships^8,44–46^, and prior studies examined whether these behaviors were negatively affected during the COVID-19 pandemic due to social restriction measures. Whereas empathy is more sensitive to individual differences^47^, prosocial behavior shows developmental changes in the teenage years^8,46,48,49^. A study on the effects of pandemic measures on empathy and prosocial behavior showed that early in the pandemic (4 weeks in the pandemic) these behaviors were negatively affected, possibly reflecting fewer social opportunities and a focus on the self rather than others^50^. However, another study reported that prosocial behavior increased with pandemic length, possibly reflecting coping or a need to contribute to society^51^. Therefore, the present study tests the effect of pandemic length on brain development as well as empathy and prosocial behavior.

The aim of the present study was to examine the effects of the COVID-19 pandemic on teenage children using a unique ongoing longitudinal study. This study is part of the longitudinal L-CID study^52^ in which twin participants were included in 2015 and tested annually with behavioral measures and bi-annually MRI measures. The COVID-19 pandemic started during data collection of “wave 5” of the L-CID study when approximately half of the participants were tested before the 13th of March 2020 and the other half was tested in the period of the 25th of July 2020 to the 28th of April 2021, during the COVID-19 pandemic. With matching by age, it was possible to select two subgroups of which approximately half was tested before (*before*-pandemic group, n = 114) and the other half tested during (*peri*-pandemic group, n = 204) the COVID-19 pandemic. The groups were comparable on all other aspects of the study. In this region-of-interest study we hypothesized that the COVID-19 pandemic related experiences would accelerate social brain development by larger decreases in grey matter thickness and surface area in the *peri*-pandemic compared to the *before*-pandemic group^24^. In addition, we tested for effects of pandemic duration in the *peri*-pandemic group to investigate whether effects were leveling off or increasing with longer duration of the pandemic^43^. We examined similar effects within the amygdala and hippocampus based on prior studies showing that amygdala growth mainly accelerates during stressful events in childhood^33,39^ and hippocampal volume growth either accelerates^39^ or shows to be stable with a possible delayed effect of stress later in adulthood^30^. Finally, we examined effects of pandemic duration on brain development, empathy and prosocial behavior. Given the inconsistent findings in prior studies, we had no hypotheses for the direction of these effects.

## Methods

### Participants

The present study is part of the Longitudinal Leiden Consortium on Individual Development (L-CID) twin study^52^. The children were same-sex twins born between 2006 and 2009 and recruited through municipal registries^53^. The study was approved by the Dutch Central Committee on Research Involving Human Subjects (CCMO) and all research was performed in accordance with relevant guidelines and regulations. DNA analyses through buccal cell samples, that were collected with mouth swabs (Whatman Sterile Omni Swab), were used to determine the zygosity of the twin pairs. The families were living in the western part of the Netherlands and all twin pairs had a shared environment at home. Furthermore, the participants spoke Dutch fluently, reported normal or corrected-to-normal vision, and reported no neurological or psychiatric impairments. Children participated in up to three biennial MRI assessments and 6 annual behavioral assessments (Crone et al., 2020). Prior to the first two MRI visits, informed consent was obtained from both parents. At the third MRI visit, the children also provided a signed informed consent. The family, including the parent that spends the most time with the twin pair, were asked to partake in the lab visit at the Leiden University Medical Center (LUMC).

The present study involved a total of 467 participants (aged between 9 and 13 years old; 51% female) that took part in “wave 3” and/or “wave 5” of data collection. For the purpose of the present study, we refer from now on to “wave 3” as timepoint 1 and “wave 5” as timepoint 2. At time point 1 (i.e., “wave 3”), 456 participants were included (9–11 years old). At time point 2 (i.e., “wave 5”) 336 participated in the study (11–13 years old).

### Pandemic timeline

**Figure 1:**
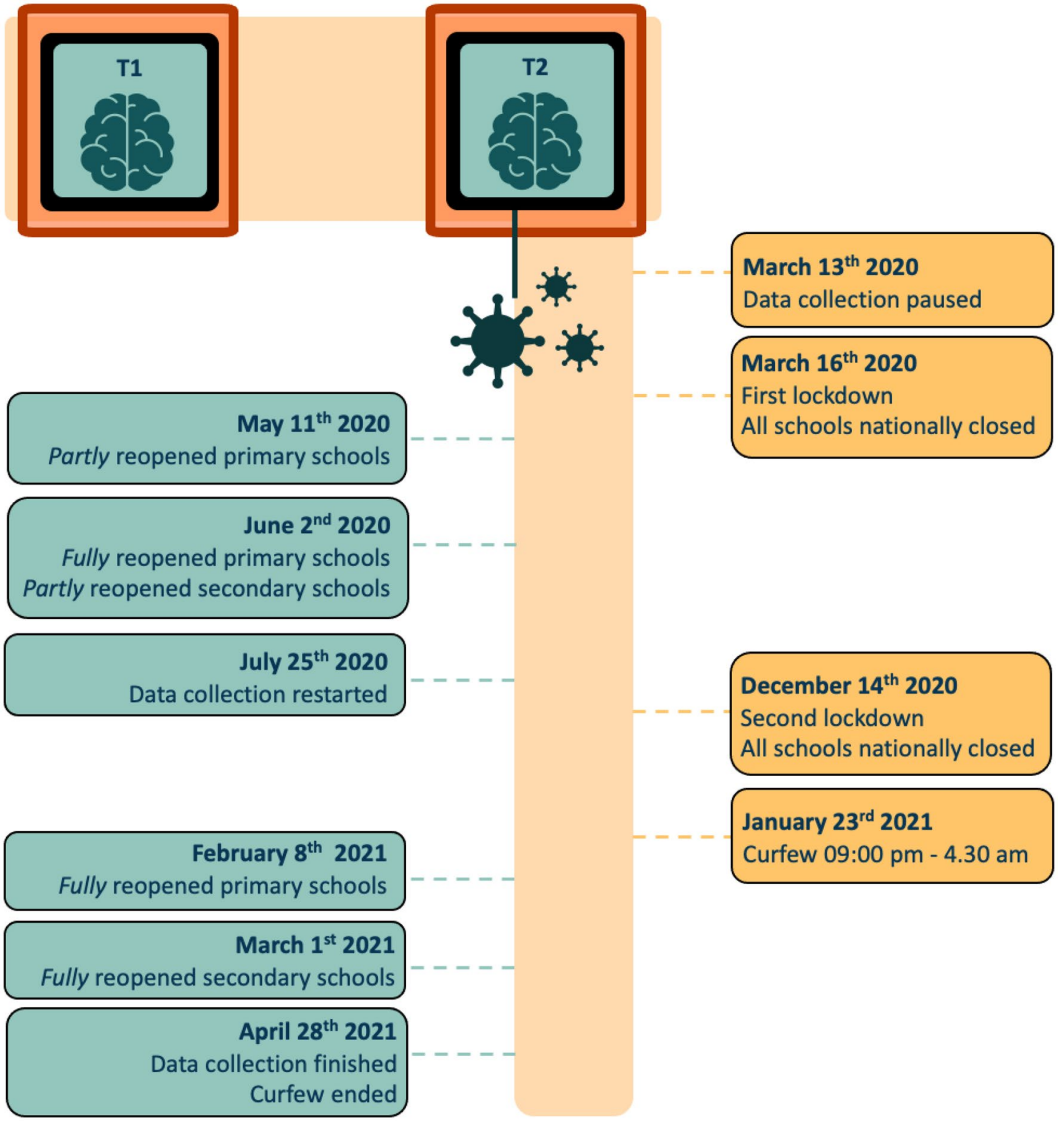
Timeline of COVID-19 pandemic measures in the Netherlands during data collection of MRI time point 2. Note that the strict restrictions of the COVID-19 pandemic are depicted in orange on the right and the more lenient restrictions are depicted in turquoise on the left. T1 = time point 1; T2 = Time point 2.

During data collection of MRI time point 2 (“wave 5”;^52^, the Netherlands was involved in a lockdown due to the COVID-19 pandemic that started on March 16th 2020^54^. As a result of the lockdown measures, all schools were nationally closed (see Fig. 1 for the timeline of the COVID-19 pandemic measures in the Netherlands and our data collection). On May 11th 2020, the government partly reopened the primary schools and fully opened on June 2nd 2020. In addition, secondary schools were partly reopened on 2nd of June 2020 as well. Other lockdown measures remained in place (e.g., a maximum amount of three home visitors). Since the number of infections rapidly increased in the period hereafter, the government announced a second lockdown at December 14th 2020. Again, all schools were nationally closed. Another measure in the second lockdown was added on January 23rd 2021, requiring Dutch citizens to be at home on time for the curfew that was nationally set from 9:00 pm to 4.30 am. On February 8th 2021, the government reopened primary schools whereas secondary schools were reopened on March 1st 2021. Researchers were obligated to pause the data collection due to lockdown measures at the LUMC on March 13th 2020. By that time, we collected data of 114 participants in the *before-*pandemic group. On July 25th 2020, we restarted the data collection of time point 2^52^. Hence, we collected data of the remaining 222 participants in the *peri*-pandemic group. Data collection of timepoint 2 was completed on the 28th of April in 2021 (total n = 336). The intervention of the COVID-19 pandemic allowed us to compare behavioral changes and structural brain development between the participants in the *before*-and *peri-*pandemic groups, and to examine the effects of duration of pandemic length.

To make sure the groups did not differ on age, we matched the two groups on age and solely included participants aged between 11.1 and 13.7 at time point 2. As such, 18 participants were excluded from the *peri*-pandemic group in subsequent analyses. The behavioral sample at time point 1 consisted of 285 participants and 292 at time point 2. The MRI sample consisted of 255 participants at time point 1 and 223 at time point 2. See Table 1 for the demographic characteristics per group. At wave 1 in 2015^52^, we asked both caregivers for their education levels as a measure of parental socio-economic status (SES) (levels: low, middle, and high). Low SES involved parents that both completed vocational education at most, whereas high SES involved parents that completed at least preparatory college education. Middle SES included the remaining combinations of education levels of both parents. Furthermore, intelligence quotient (IQ) was also measured at wave 1, based on subtests “similarities” and “block design” of the WISC (3rd edition; Kaufman, 1994). Finally, sex was based on birth record reports. These demographic characteristics were compared between the *before-* and *peri-*pandemic group using t-tests for continuous data and Chi-Square Goodness of Fit Tests for categorical data. A Mann–Whitney U t-test indicated that the *before*- and *peri*-pandemic groups did not differ in age on time point 1 (*W* = 13,938, *p* = 0.05). Independent t-tests indicated that *before*- and *peri*-pandemic groups did not differ in age on time point 2 (*t*(241) = − 1.70, *p* = 0.09 and IQ (*t*(241) = − 1.87, *p* = 0.06). Furthermore, Chi-Square Goodness of Fit Tests were performed to determine whether the proportions of sex, SES, and zygosity were equal between the *before*-and *peri-*pandemic group. The proportions in the groups did not differ by sex (*X*^2^(1, 328) < 0.001, *p* = 0.99), SES (*X*^2^ (2, 328) = 2.13, *p* = 0.34), or zygosity (*X*^2^(1, 328) = 0.27, *p* = 0.61).

**Table 1:** Demographic characteristics for each pandemic group.

	Before-pandemic	Peri-pandemic
*N*	114	204
Boys	49%	49%
Monozygotic	57%	55%
Left-handed	13%	14%
Age time point 1 (*SD*)	10.08 (.65)	9.79 (.61)
Age time point 2 (*SD*)	12.15 (.67)	12.29 (.72)
Range	8.99–13.34	9.03–13.63
SES: low, middle,high (%)^a^	1.8–47.4–50.9	4.90–47.1–47.1
Median IQ^b^	105	105
IQ range	72.5–132.5	77.5–137.5

*N* number of participants, *SD* standard deviation.

^a^Socio-economic status (SES), based on parental education at wave 1.

^b^Intelligence quotient (IQ), based on subtests “similarities” and “block design” of the WISC (3rd edition) at wave 1.

### MRI data acquisition

The procedures of all acquired scans were similar during the *before*- and *peri*-pandemic lockdown, with the exception that extra hygiene measures (e.g., hand washing and wearing masks) were taken in the *peri*-group. Prior to each scan session, participants were familiarized with the scan protocol to reduce the potential of high emotional arousal and motion^56^. Next, the official scan protocol started where MRI scans were acquired on a Philips Ingenia 3.0 Tesla MRI system at the LUMC. Here, a standard whole-brain coil was used including foam inserts next to both ears, limiting participants’ head movement during the MRI session. As anatomical scan of interest for each participant, high-resolution 3D T1-weighted scans were obtained using the following settings: FOV = 224 (ap) × 177 (rl) × 168 (fh); TR = 9.72 ms; TE = 4.95 ms; FA = 8°; 140 slices; voxel size 0.875 × 0.875 × 0.875 mm. During the T1-weighted scan acquisition, participants watched a movie through a small mirror attached to the head coil to limit possible head motion. After the acquisition, the scan was manually checked for excessive motion (e.g., visible movement rings). When time allowed, the T1-weighted scan acquisition was repeated if excessive motion was observed.

### MRI processing

The processing of the T1-weighted scans was performed using the validated FreeSurfer software (v7.1.1; https://surfer.nmr.mgh.harvard.edu/). FreeSurfer allowed to label tissue and regional classification and includes tools to carry out volume-and-surface based analyses. Several automated processing steps are included in the first processing pipeline, such as gray matter segmentation^57–59^, non-brain tissue elimination^60^, boundary of gray and white matter corrections^61^ and intensity normalization^62^. As a next step, a longitudinal processing pipeline was used to decrease within-subject variability between the sessions in our longitudinal design and to increase statistical power^63,64^.

### Quality control

After MRI processing, the quality of the T1-weigthed scans were manually determined using the Quala-T tool including a set of quality criteria (e.g., incorrect removal of non-brain tissue, missing brain regions during reconstruction, and excessive head motion). For more details of the protocol procedure see^65^). Based on these criteria, it was determined by three trained raters whether cortical reconstruction was sufficiently acceptable for each of the scans.

### Regions of interest analyses

**Figure 2:**
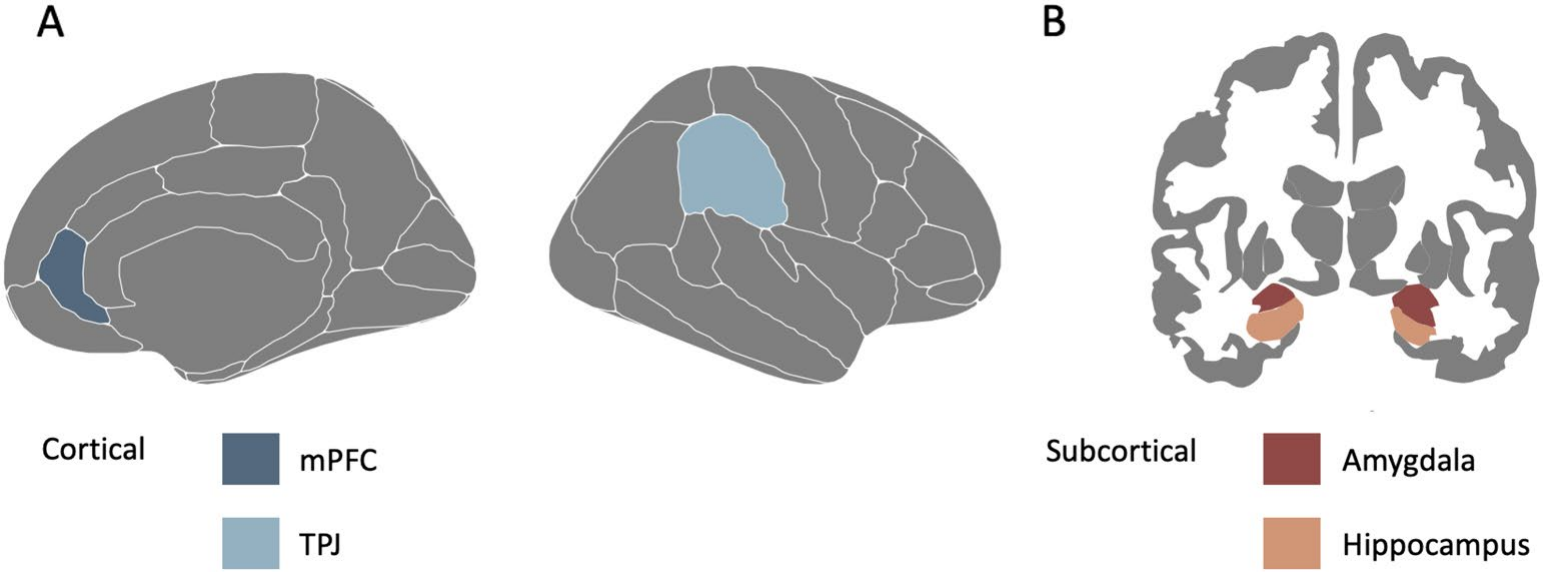
An overview of the ROIs used for neural analyses with in (**A**) cortical (social network) and in (**B**) subcortical (stress network) brain regions. In total 4 ROIs are presented in the figure. mPFC = medial prefrontal cortex, TPJ = temporoparietal junction.

Brain regions of interest (ROIs) were selected based on prior studies involved in the social and stress network^6,7,22,29,66^. Specifically, the following bilateral brain regions of the stress network were included: amygdala and hippocampus (FreeSurfer Fischl atlas:^57^). Additionally, the following bilateral brain regions of the social network were selected: mPFC (rostral anterior cingulate cortex) and TPJ (supramarginal cortex) (FreeSurfer Desikan-Killiany atlas:^67^), given that these regions in prior research showed large sensitivity to environmental experiences^21,22^. See Fig. 2 for an overview of the ROIs in the stress and social network. On a cortical level, we reported on thickness and surface area, whereas on a subcortical level on volume. We combined structural measures of both hemispheres. As such, we controlled for the size of each brain region including surface area (SA) for bilateral cortical thickness (CT) by computing: $\frac{\left(rhCT*rhSA\right) + (lhCT*lhSA)}{(rhSA + lhSA)}$*.* We computed the mean of the right (rh) and left hemisphere (lh) for bilateral volume (VO) and SA, using the following formula: $\frac{(rhVO + lhVO)}{2}$ or $\frac{(rhSA + lhSA)}{2}$.

### Parent-reported prosocial behavior

Prosocial behavior was measured using two different parent-reported questionnaires including the 13-item “Emphatic and prosocial response to another distress” subscale of the My Child Questionnaire (MC;^68^) and the 5-item “Prosocial” subscale of the Strengths and Difficulties Questionnaire (SDQ;^69^. These questionnaires were both administered time point 1 and time point 2 of the middle childhood cohort, and show acceptable reliability^52,68,70^. The MC subscale consisted of a 5-point Likert scale ranging from 0 = “not true” to 4 = “true” and involves items such as “My child can tell at just a glance how others are feeling”. The SDQ subscale consisted of a 3-point Likert scale ranging from 0 = “not true” to 2 = “certainly true” and involves items such as “My child often volunteers to help others”. The total of 18 items of both questionnaires were factor analyzed by computing a principal component analysis (PCA) using Varimax rotation^22^. In order to compare the scores of the SDQ (ranging from 1 to 3) with the MC (ranging from 1 to 5), we first recoded the SDQ scores from 0–1–2 to 0–2–4. Additionally, one of each twin pair was randomly divided over two samples (A and B) to prevent within-twin clustering in the PCA analysis. As such, one of the twin pair was assigned to sample A whereas the other co-twin was assigned to sample B.

As a first step, we computed the PCA in sample A of time point 1^52^ using only items of one of the parents. This analysis resulted in two components, “Prosocial” and “Empathy”, where KMO (0.80) and Bartlett’s test (*X*^2^ (153) = 746.31, *p* < 0.001) both revealed that all 18 items could be used in the PCA analysis. Here, “Prosocial” explained 26.15% of the variance and “Empathy” explained 13.11% of the variance. Two items were excluded for further analyses, including “My child feels good when good things happen to movie characters” and “My child may occasionally tease a pet if unsupervised” (recoded), because they did not fit well in any of the created factors (factor loading < 0.3). The PCA analyses were repeated for the items of the other parent in sample A and the items of both parents in sample B. In all samples including time point 2^52^, similar outcomes of the components were observed. As a final step, the mean of the items that were part of the two new created factors were computed and used as subscales of “Prosocial behavior” and “Empathy”. Herewith, a higher score indicated more empathy or prosocial behavior. Since the correlations between both parents were significantly positive for both subscales (Prosocial Behavior: sample A: r = 0.48; sample B: r = 0.53, *p*’s < 0.001; Empathy: sample A: r = 0.37; sample B: r = 0.43, *p*’s < 0.001), the mean of the ratings of both parents were calculated for both subscales and used for subsequent analyses.

### Statistical analyses

Linear mixed-effects models were used to investigate the effect of pandemic lockdown (*before* and *peri*) on brain development of the social (i.e., cortical thickness and surface area of the mPFC and TPJ) and stress network (i.e., subcortical volume of the hippocampus and amygdala). In addition, we investigated the effects of pandemic lockdown on behavioral development of prosociality and empathy. We did so by using the lme4 package^71^ in R (Team, 2015) and inspected the results with the type III ANOVA’s Satterthwaite’s method^73^. Whenever significant main effects were observed we inspected post hoc results using least-square means with Kenward–Roger corrected degrees of freedom and Bonferroni-adjusted *p*-values. In our analyses, we included random intercepts of the child and family to account for the nesting effects between twin pairs within families (ChildID and FamilyID). The fixed effects consisted of time point (time point 1, time point 2), pandemic group (before, peri) and sex (male, female). All main effects and the two-way interactions were obtained (time point x pandemic). To examine brain and behavioral development, we specified the fitted linear mixed model in R as:

$${\sum }_{i=1}^{N}Brain/Behavior\sim Time\,point \times pandemic+ sex+ \left(1|ChildID\right)+ \left(1|FamilyID\right) + \varepsilon$$

Additionally, we tested in the *peri*-pandemic group whether individual differences in structural brain change of the ROIs and changes of prosocial and empathic behavior between time point 1 and 2 were associated with differences in length of pandemic using Pearson correlation tests. The pandemic length is defined as the difference time between the *date of the lab visit* and the *start of the lockdown* (15th of March 2020) in days. Individual rates of change of the brain ROIs and behavioral measures between time point 2 and 1 were controlled for age and based on complete cases of two time points, and specified in R as:

$${\sum }_{i=1}^{N}Rate\,of\,change =\frac{(ROI\,or\,behavior\,time\,point\,2-ROI\,or\,behavior\,time\,point\,1)}{(Age\,time\,point\,2-Age\,time\,point\,1)}$$

We used a Bonferroni correction for multiple testing in our neural and behavioral analyses, adjusted for correlated variables^74,75^; http://www.quantitativeskills.com/sisa/calculations/bonfer.htm). The average correlation between the cortical thickness measures was r = 0.25, the surface area measures was r = 0.45, volume measures was r = 0.70, and behavioral measures was r = 0.36. This resulted in adjusted significance criteria of *α* (2-sided adjusted) = 0.036 for cortical thickness, *α* (2-sided adjusted) = 0.031 for surface area, and *α* (2-sided adjusted) = 0.043 for subcortical volume analyses. We reported all significant predictors for transparency based on the significance level of *p* < 0.05 and additionally mentioned whether it survived Bonferroni correction.

### Ethics approval

The study and procedures were approved by the Dutch Central Committee on Research Involving Human Subjects (CCMO) and all research was performed in accordance with relevant guidelines and regulations.

## Results

### Control analyses

Table 2 shows the means and standard deviations (SDs) for each brain and behavior measure at both time points per pandemic group. First, we tested whether pandemic groups differed on the dependent variables on the first time point. As can be seen in Table 2, only for TPJ thickness we observed that the means differed between the *before*- and *peri*-pandemic group on time point 1 (*t*(211) = 2.76, *p* = 0.006). Here, relatively higher TPJ thickness was observed in the *before* compared to the *peri*-pandemic group at time point 1. For all other measures, values did not differ at the first time point (all *p*’s > 0.15). As a sensitivity check, we controlled for age on time point 1 in all the subsequent linear mixed models and all effects remained significant (see Methods for statistical approach).

**Table 2:** Means and standard deviations of the dependent variables for each time point per group.

	*Before*-pandemic T1 (wave 3)	*Before*-pandemic T2 (wave 5)	*Peri*-pandemic T1 (wave 3)	*Peri*-pandemic T2 (wave 5)
	M (*SD*)	M (*SD*)	M (*SD*)	M (*SD*)
mPFC cortical thickness	3.19 (.12)	3.14 (.13)	3.20 (.14)	3.14 (.14)
TPJ cortical thickness	2.97 (.09)**	2.91 (.09)	2.93 (0.10)**	2.87 (.10)
mPFC surface area	746.42 (126.51)	749.21 (132.77)	745.20 (134.98)	756.49 (140.78)
TPJ surface area	4074 (611.55)	4009.46 (606.80)	4137.58 (645.90)	4142.55 (658.33)
Hippocampus volume	4278.39 (414.77)	4345.39 (402.45)*	4347.04 (412.46)	4482.30 (414.10)*
Amygdala volume	1759.94 (184.93)	1789.42 (202.03)	1772.11 (208.62)	1819.52 (211.68)
Prosocial behavior	3.37 (.39)	3.44 (.44)	3.33 (.15)	3.39 (.49)
Empathy behavior	2.05 (.72)	1.99 (.83)	2.19 (.80)	2.06 (.88)

*M* mean, *SD* standard deviation, *mPFC* medial prefrontal cortex, *TPJ* temporoparietal junction, Control analyses showed that TPJ cortical thickness differed between the before and peri pandemic group at time point 1. Hippocampus volume differed between the before and peri pandemic group at time point 2; *Significance of *p* < .05; **Significance of *p* < .01.

### Effects of pandemic on structural brain development

**Figure 3:**
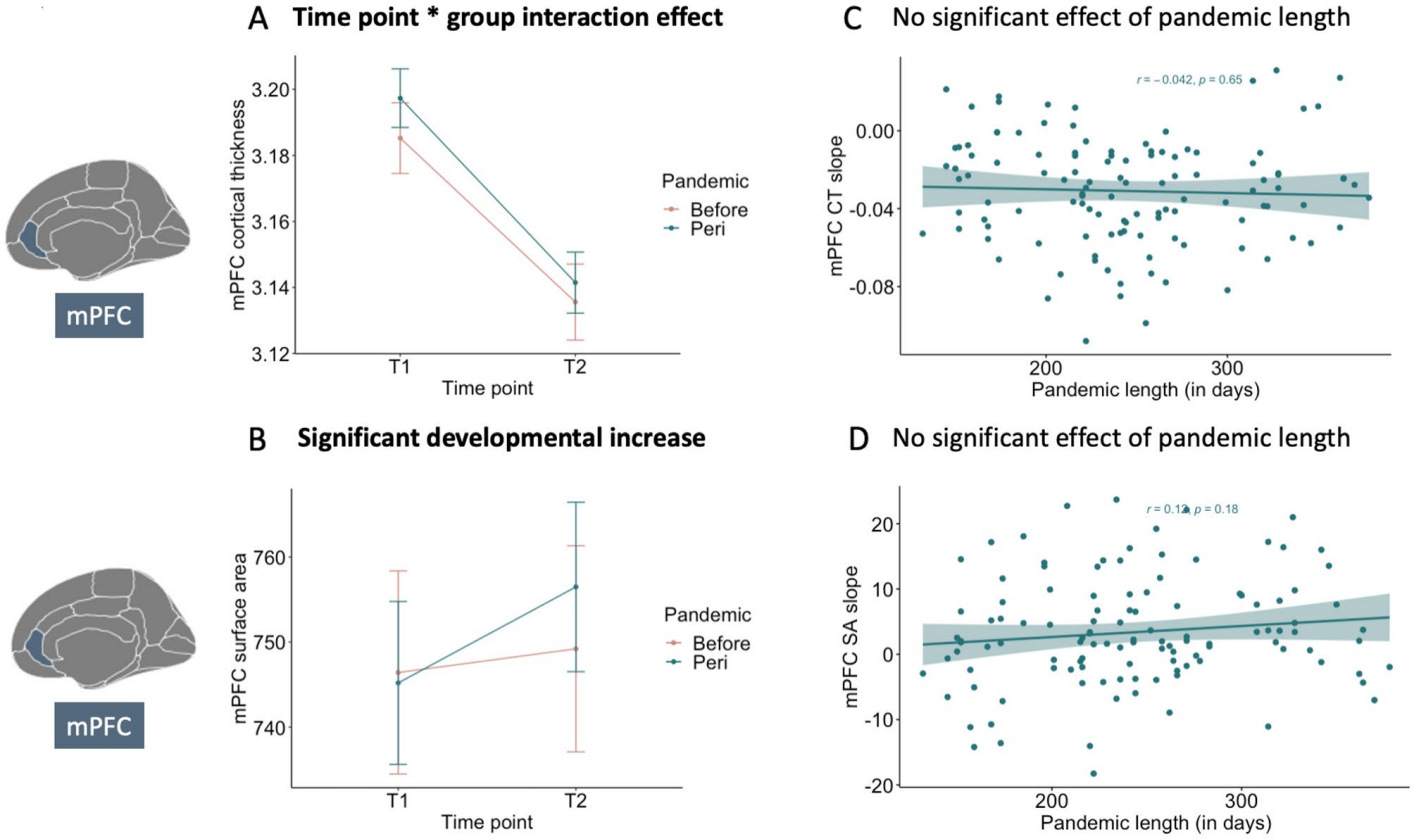
Visualizations of the linear mixed model results of mPFC thickness and surface area development. In (**A**) mPFC cortical thickness is presented on the y-axis and time point on the x-axis. Note that the *peri*-pandemic group showed accelerated decrease of development. In (**B**) mPFC surface area is presented on the y-axis and time point on the x-axis, showing a significant developmental increase independent of pandemic group. In (**C**) slopes of mPFC thickness of participants in the *peri*-pandemic group are presented on the y-axis and pandemic length (in days) on the x-axis, showing no association. In (**D**) slopes of mPFC surface area of participants in the *peri*-pandemic group are presented on the y-axis and pandemic length (in days) on the x-axis, showing no association. mPFC = medial prefrontal cortex; *CT* cortical thickness, *SA* surface area.

Six linear mixed models were performed to determine whether cortical thickness of the mPFC and TPJ, surface area of mPFC and TPJ, and volume of hippocampus and amygdala brain regions differed between the two time points and whether it showed an interaction effect with pandemic group, with sex as additional covariate, and random intercepts for the child and family. Results showed that mPFC thickness significantly decreased between the time points (*F*(1, 195) = 181.19, *p* < 0.001), where relatively higher mPFC thickness was observed at time point 1 (*M* = 3.20 mm, *SE* = 0.01, 95% CI [3.18, 3.22]) compared to time point 2 (*M* = 3.13, *SE* = 0.01, 95% CI [3.11, 3.15]). In addition, we observed an interaction effect of time point and pandemic group (*F*(1, 195) = 4.08, *p* = 0.044), where results indicated a relatively larger decrease of mPFC cortical thickness in the *peri* (*b* = 0.07) compared to the *before* (*b* = 0.05) group. However, this interaction effect did not survive Bonferroni correction. See Fig. 3A for the pandemic effect on mPFC thickness development.

Results indicated that mPFC surface area significantly increased between the two time points (*F*(1, 189) = 17.04, *p* < 0.001), where lower mPFC surface area was observed at time point 1 (*M* = 742 mm, *SE* = 9.59, 95% CI [723, 761]) compared to time point 2 (*M* = 749 mm, *SE* = 9.61, 95% CI [730, 768]). No interaction effect was observed between time point and pandemic group (*F*(1, 189) = 0.80, *p* = 0.37). Furthermore, we observed a main effect of sex (*F*(1, 151) = 13.43, *p* < 0.001), where boys showed relatively higher mPFC surface area (*M* = 779 mm, *SE* = 13.4, 95% CI [752, 805]) compared to girls (*M* = 712 mm, *SE* = 13.1, 95% CI [687, 738]), but sex did not interact with timepoint of pandemic group (*F*(1, 189) = 1.71, *p* = 0.19). See Fig. 3B for the significant developmental increase of mPFC surface area independent of pandemic effects.

**Figure 4:**
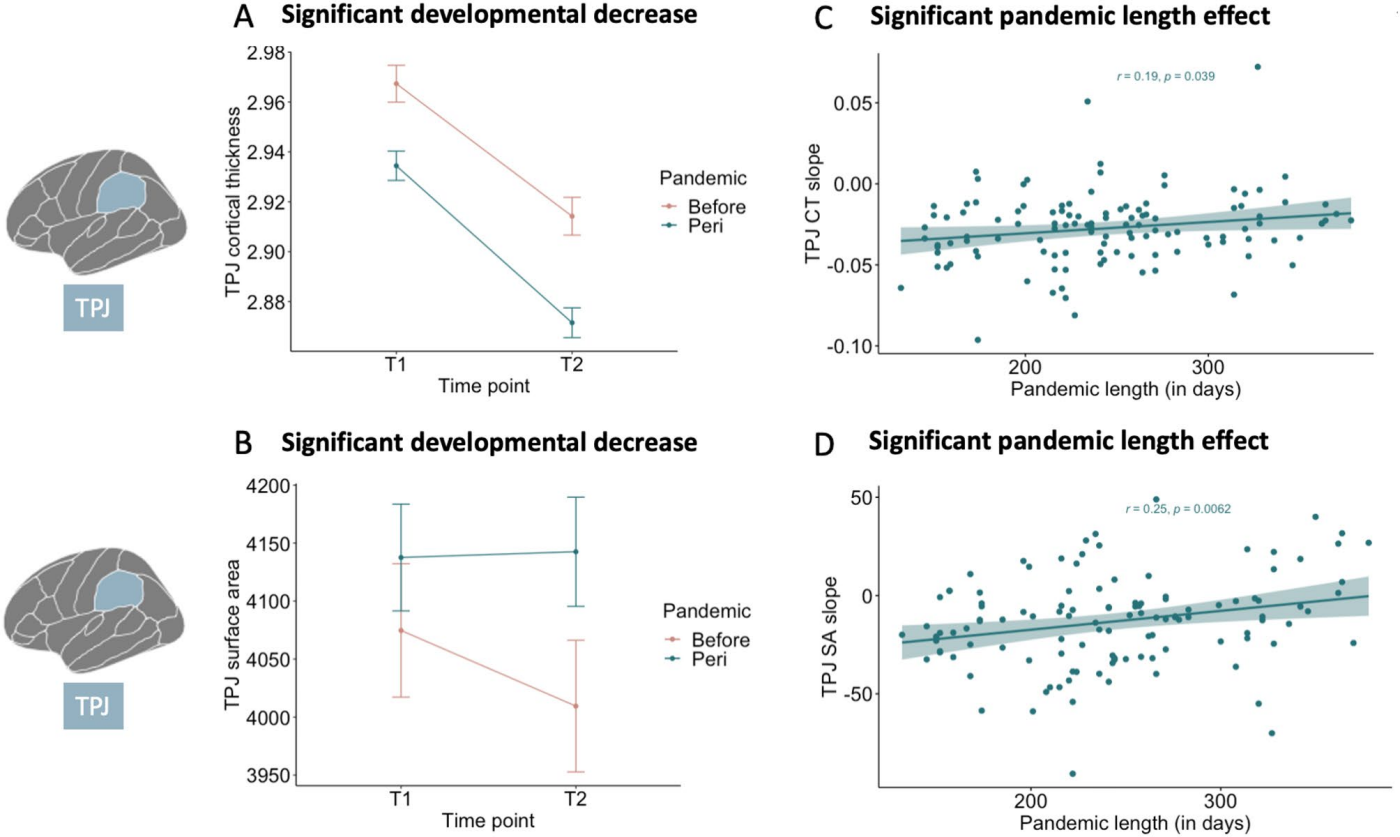
Visualizations of the linear mixed model results of TPJ thickness and surface area development. In (**A**) TPJ cortical thickness is presented on the y-axis and time point on the x-axis, showing a significant developmental decrease independent of pandemic group. In (**B**) TPJ surface area is presented on the y-axis and time point on the x-axis, showing a significant developmental decrease independent of pandemic group. In (**C**) slopes of TPJ cortical thickness of participants in the *peri*-pandemic group are presented on the y-axis and pandemic length (in days) on the x-axis, showing a positive association. In (**D**) slopes of mPFC surface area of participants in the *peri*-pandemic group are presented on the y-axis and pandemic length (in days) on the x-axis, showing a positive association. *TPJ* temporoparietal junction; *CT* cortical thickness, *SA* surface area.

Results showed that TPJ thickness also significantly decreased between the two time points (*F*(1, 201) = 277.88, *p* < 0.001), where relatively higher TPJ thickness was observed at time point 1 (*M* = 2.95 mm, *SE* = 0.01, 95% CI [2.94, 2.97]) compared to time point 2 (*M* = 2.89 mm, *SE* = 0.01, 95% CI [2.88, 2.90]). No interaction effect of time point and pandemic group was observed (*F*(1, 201 = 0.87, *p* = 0.35). See Fig. 4A for the significant negative slopes (i.e., developmental decrease) of TPJ thickness independent of pandemic effects.

Results indicated that TPJ surface area significantly decreased between the two time points (*F*(1, 187) = 57.13, *p* < 0.001), where relatively higher TPJ surface area was observed at time point 1 (*M* = 4104 mm, *SE* = 41.6, 95% CI [4022, 4186]) compared to time point 2 (*M* = 4072 mm, *SE* = 41.6, 95% CI [3990, 4154]). No interaction effect was observed between time point and pandemic group (*F*(1, 187) = 0.01, *p* = 0.94). Furthermore, a main effect of sex was observed (*F*(1, 149) = 33.13, *p* < 0.001), where boys showed relatively higher TPJ surface area (*M* = 4314 mm, *SE* = 58.1, 95% CI [4200, 4429]) compared to girls (*M* = 3861 mm, *SE* = 56.9, 95% CI [3749, 3974]), but sex did not interact with timepoint of pandemic group (*F*(1, 187) = 0.60, *p* = 0.44). See Fig. 4B for the for the significant negative slopes (i.e., developmental decrease) of TPJ surface area independent of pandemic effects.

**Figure 5:**
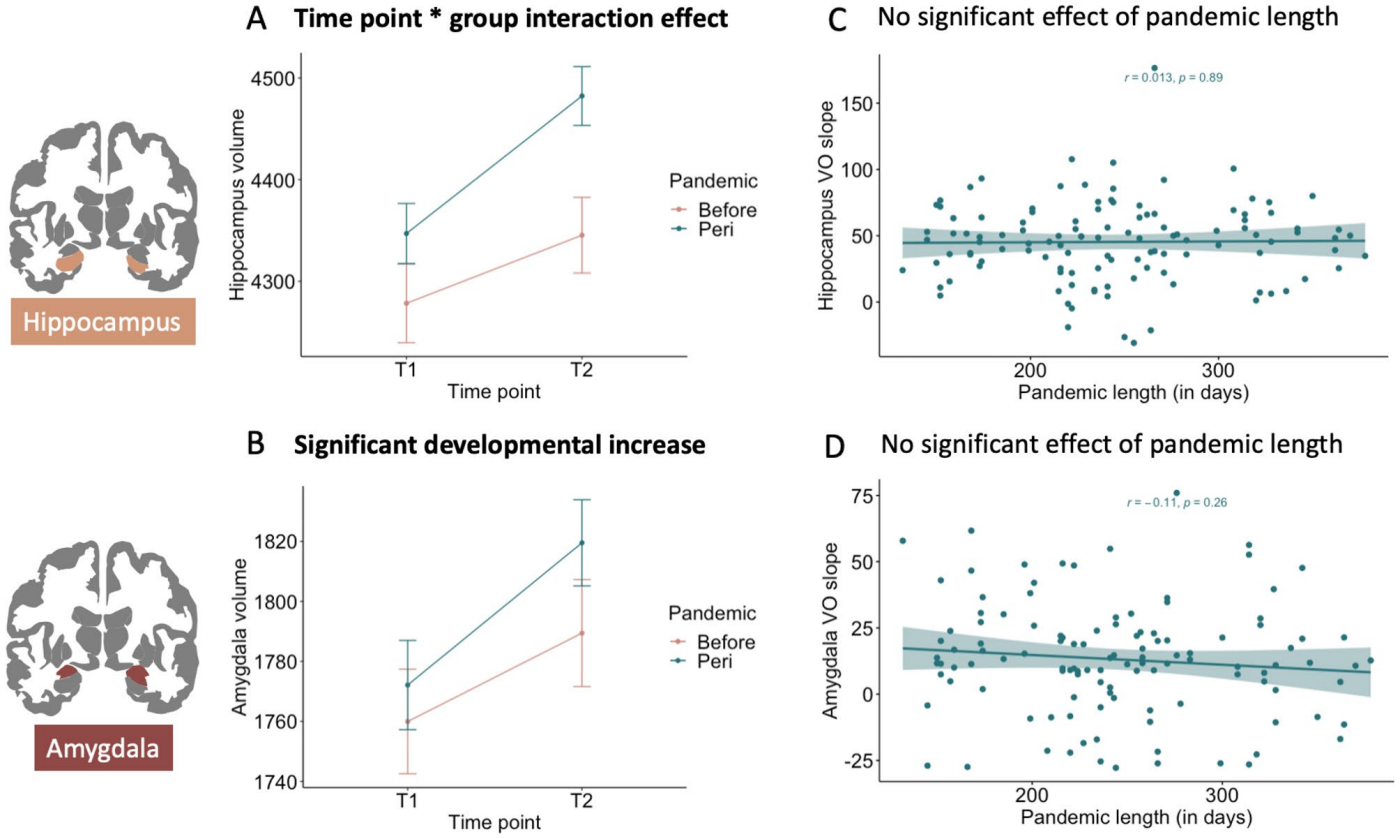
Visualizations of the linear mixed model results of volumetric hippocampus and amygdala development. In (**A**) hippocampus volume is presented on the y-axis and time point on the x-axis. Note that the *peri*-pandemic group showed accelerated increase of hippocampal development. In (**B**) amygdala volume is presented on the y-axis and time point on the x-axis, showing a significant developmental increase independent of pandemic group. In (**C**) slopes of volumetric hippocampus of participants in the *peri*-pandemic group are presented on the y-axis and pandemic length (in days) on the x-axis, showing no association. In (**D**) slopes of volumetric amygdala of participants in the *peri*-pandemic group are presented on the y-axis and pandemic length (in days) on the x-axis, showing no association. *VO* volume.

Results showed that hippocampus volume significantly increased between the two time points (*F*(1, 190) = 343.88, *p* < 0.001), where lower hippocampus volume was observed at time point 1 (*M* = 4315 mm, *SE* = 28.7, 95% CI [4258, 4372]) compared to time point 2 (*M* = 4416 mm, *SE* = 28.8, 95% CI [4359, 4473]). Additionally, we observed an interaction effect between time point and pandemic group (*F*(1, 190) = 6.21, *p*_*corrected*_ < 0.04), where results indicated a relatively larger increase of hippocampus volume in the *peri* (*b* = -114.4) compared to the *before* (*b* = − 87.3) group. Finally, results indicated a main effect of sex (*F*(1, 150) = 44.69, *p* < 0.001), where boys showed relatively higher hippocampus volume (*M* = 4547 mm, *SE* = 40.0, 95% CI [4468, 4626]) compared to girls (*M* = 4184 mm, *SE* = 39.2, 95% CI [4107, 4262]), but sex did not interact with timepoint of pandemic group (*F*(1, 190) = 0.0004, *p* = 0.98). See Fig. 5A for the pandemic effect on volumetric hippocampus development.

Results showed that amygdala volume significantly increased between the two time points (*F*(1, 192) = 65.15, *p* < 0.001) , where lower amygdala volume was observed at time point 1 (*M* = 1768 mm, *SE* = 14.1, 95% CI [1741, 1796]) compared to time point 2 (*M* = 1801 mm, *SE* = 14.2, 95% CI [1773, 1829]). No interaction effect was observed between time point and pandemic group (*F*(1, 192) = 0.30, *p* = 0.58). Furthermore, a main effect of sex was observed (*F*(1, 152) = 42.37, *p* < 0.001), where boys showed relatively higher amygdala volume (*M* = 1871 mm, *SE* = 19.6, 95% CI [1832, 1910]) compared to girls (*M* = 1698 mm, *SE* = 19.2, 95% CI [1660, 1736]), but sex did not interact with timepoint of pandemic group (*F*(1, 192) = 0.72, *p* = 0.39). See Fig. 5B for the significant positive slopes (i.e., developmental increase) in volumetric amygdala development independent of pandemic effects.

### Effects of pandemic on prosocial and empathic behavior

**Figure 6:**
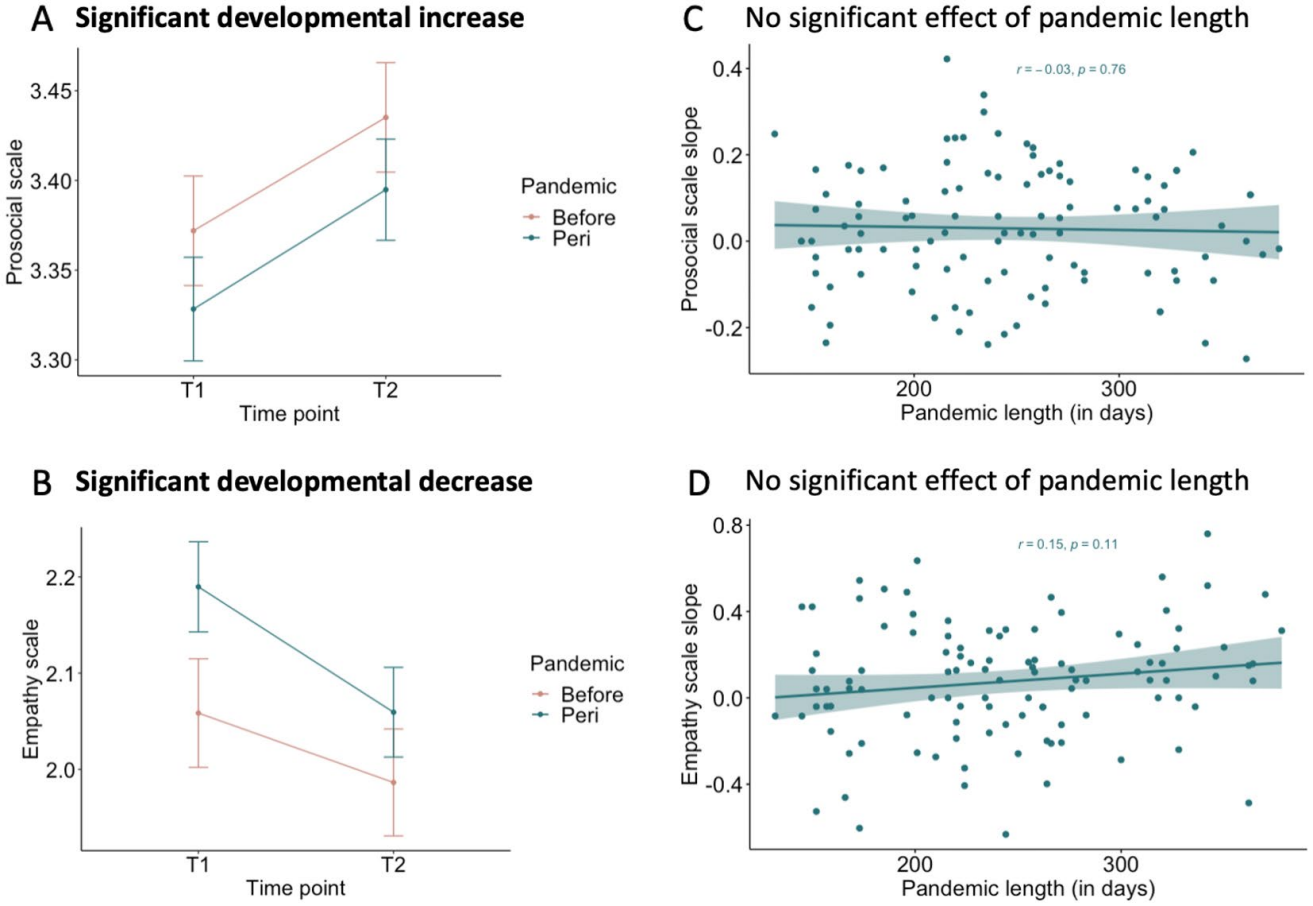
Visualizations of the linear mixed model results of prosocial and empathic development. In (**A**) prosocial behavior scale is presented on the y-axis and time point on the x-axis, showing a significant developmental increase independent of pandemic group. In (**B**) empathic behavior scale is presented on the y-axis and time point on the x-axis, showing a significant developmental decrease independent of pandemic group. In (**C**) slopes of prosocial behavior of participants in the *peri*-pandemic group are presented on the y-axis and pandemic length (in days) on the x-axis, showing no association. In (**D**) slopes of empathic behavior of participants in the *peri*-pandemic group are presented on the y-axis and pandemic length (in days) on the x-axis, showing no association.

Two linear mixed models were performed to determine whether emphatic and prosocial behavior differed between the two time points and whether it showed an interaction effect with pandemic group. Results showed that prosocial behavior significantly differed between time points, *F*(1, 281) = 8.41, *p* = 0.004), where lower prosocial scores were observed at time point 1 (*M* = 3.35, *SE* = 0.03, 95% CI [3.29, 3.41]) compared to time point 2 (*M* = 3.41, *SE* = 0.03, 95% CI [3.35, 3.47]). However, time point did not significantly interact with pandemic group (*F*(1, 281) = 0.01, *p* = 0.91). A main effect of sex was observed (*F*(1, 155) = 18.32, *p* < 0.001), where the highest prosocial scores were observed in girls (*M* = 3.49, *SE* = 0.04, 95% CI [3.42, 3.57]) compared to boys (*M* = 3.27, *SE* = 0.04, 95% CI [3.19, 3.34]), but sex did not interact with timepoint of pandemic group (*F*(1, 286) = 1.76, *p* = 0.19). See Fig. 6A for the developmental increase of prosocial behavior independent of pandemic effects.

Results showed that empathic behavior significantly differed between time point 1 and 2 (*F*(1, 287) = 9.25, *p* = 0.002), where higher empathy scores were observed at time point 1 (*M* = 2.12, *SE* = 0.06, 95% CI [2.01, 2.23]) compared to time point 2 (*M* = 2.00, *SE* = 0.06, 95% CI [1.89, 2.12]). No significant interaction effect was observed between time point and pandemic group (*F*(1, 287) = 0.93, *p* = 0.34). Furthermore, a main effect of sex was observed (*F*(1, 151) = 27.94, *p* < 0.001), where relatively higher empathy scores were observed in girls (*M* = 2.33, *SE* = 0.07, 95% CI [2.19, 2.48]) compared to boys (*M* = 1.79, *SE* = 0.08, 95% CI [1.64, 1.94]), but sex did not interact with timepoint of pandemic group (*F*(1, 281) = 0.12, *p* = 0.73). See Fig. 6B for the developmental decrease of empathic behavior independent of pandemic effects.

### Brain and behavioral development with pandemic length association

As a next step, we examined whether individual differences in brain and prosocial/empathy change in the *peri-*pandemic group, were associated with pandemic length (in days). Both development of TPJ thickness (*r*(114) = 0.19, *p* = 0.038) and TPJ surface area (*r*(114) = 0.25, *p* = 0.006) were positively associated with pandemic length in days. However, the association of TPJ cortical thickness with pandemic length did not survive Bonferroni correction. Note that the overall developmental patterns of TPJ thickness and surface area was a negative slope. Here, increased length of pandemic (in days) was related to relatively less negative slopes of TPJ thickness and surface area rates of change. See Fig. 4C,D for the association between TPJ thickness and surface area development with pandemic length (in days). No significant associations were observed between the remaining ROIs and behavioral measures with length of pandemic (all *p*’s > 0.11; see Figs. 3C,D, 5C,D, 6C,D).

## Discussion

The aim of this study was to examine the effects of the COVID-19 pandemic related measures on brain development in cortical and subcortical regions involved in social and stress related regions. We did so by using an age-controlled longitudinal design including 9–13-year-old children of which one group was assessed *before* and the other group *during* the pandemic. The COVID-19 pandemic had unprecedented effects on social behavior across the globe including social distancing and school closings^1,2^. This study showed that brain development was related to these social and stress experiences including more pronounced cortical thinning in the mPFC in the *peri*-pandemic group relative to the *before*-pandemic control group. There was an association between pandemic length and development of TPJ thickness and surface area, showing an immediate effect that decreased over time. Pandemic length was not associated with parent-reported effects on empathy and prosocial behavior.

The question that was addressed in this study specifically focused on the effects of pandemic related measures on brain development, or slope, of cortical social brain regions and subcortical stress-related neural areas. All regions showed development-related changes that are consistent with prior studies, including a decrease in cortical thickness in mPFC and TPJ^11^, increasing surface area in mPFC and decreasing surface area in TPJ^11–13^ and increases in hippocampus and amygdala volume^26–28^. The present study showed accelerated development of cortical thinning in the mPFC in adolescents who experienced COVID-19 pandemic social restrictions relative to age-matched adolescents who were assessed before the pandemic, although these effects did not survive correction for multiple comparisons. Our findings are consistent with prior studies demonstrating that normative development of cortical thickness of the mPFC and TPJ is influenced by social experiences, such as friendship quality for the mPFC^21^ and shared environment effects in the TPJ^22^. However, prior work is not conclusive with respect to which deprived and enriched environmental experiences are associated with attenuated versus accelerated growth^42^. For instance, the participants in the present study experienced mostly social restrictions and stress which was associated with accelerated cortical development. These findings are in line with a prior review study by^24^, showing that growing up in lower SES environment is associated with accelerated cortical thinning. However, accelerated cortical thinning was also associated with higher friendship quality^21^. It is currently not well understood what the social experiences were during the pandemic and how this affected adolescents’ day-to-day experiences. Some studies reported larger tension and negative feelings during the COVID-19 pandemic^3,4,76^, whereas others reported that adolescents engaged in more online social interactions and positive media experiences^77,78^. Future studies should examine in more detail whether the observed accelerated brain developmental patterns in the present study are specifically related to negative or also possible positive social experiences due to COVID-19 related measures. Furthermore, it is unknown to what extend individuals differ in environmental susceptibility^42,79^.

We also addressed whether growing up during the COVID-19 pandemic was associated with differential development in stress-related subcortical brain regions. We observed that specifically the hippocampus showed an accelerated developmental pattern for 9–13-year-olds growing up *during* versus *before* the pandemic. This finding fits with a cross-sectional study of^39^ reporting that 16-year-old adolescents who experienced COVID-19 related pandemic effects show larger volumes of the hippocampus compared to same-aged individuals that were assessed before the pandemic*.* Other work shows inconsistent findings regarding to the effects of (chronic) stress on hippocampal development. Smaller hippocampi have been observed in adults after child maltreatment^30,80^, whereas no differences were reported in children experiencing early neglect^81,82^. Other studies reported smaller volumetric hippocampus in children living in poverty^83^ or that are exposed to parental separation^84^. The findings of the present study may suggest that volumetric alterations may depend on the chronicity and timing of stress^85^. Possibly, the participants in our sample affected by the COVID-19 pandemic restrictions may show an earlier peak in neurogenesis (reflected in increased volumetric hippocampal development) compared to the control group but at future follow-up they might show reduced hippocampal volume^24,86^. Nonetheless, it is also suggested that volumetric alteration in the hippocampus can reverse to baseline after a stress-free period due to its nature of high plasticity^85^.

Notably, no pandemic-related effects were observed in the amygdala which is inconsistent with the cross-sectional findings reported by^39^ showing larger amygdala volumes in adolescents measured during pandemic compared to adolescents that were scanned before the pandemic. While the previous study suggested that pandemic-related effects could affect amygdala development, the results of the present study indicates that amygdala volume was not affected. However, it should be noted that this prior study was a cross-sectional study whereas the current study was longitudinal, which limits direct comparisons. Furthermore, environmental effects on the amygdala previously reported by^29^ and^34^ are possibly specific to more intense or threatening social experiences such as severe deprivation or child abuse. Here, accelerated growth of volumetric amygdala was observed in children with initial exposure to chronic stress^33^. In contrast, these volumetric increases due to stress can also lead to cell death and slower amygdala development, which can subsequently result in smaller amygdala volumes in childhood^29,85,87^. Therefore, long-term exposure to severe stress in childhood may more likely be linked to relatively smaller volumetric amygdala in same-aged children compared to peers without exposure to stress^33^. These findings suggest that associations between stress experiences and amygdala structure can fluctuate by age resulting in different findings across studies.

Prior theoretical models on behavioral resilience during disasters have described that some individuals may show immediate negative consequences followed by subsequent recovery^43^. We examined whether duration of the pandemic had an accumulating or recovery effect on neural development. We observed no effects of accumulating pandemic experiences, suggesting that especially the first period of the pandemic affected brain development in mPFC and hippocampus. Even though development of the TPJ was not affected by the pandemic, we observed that longer duration of the pandemic was associated with less negative slopes of TPJ thickness and surface area. This suggests that pandemic duration might be associated with attenuated growth of the TPJ. A prior study showed that especially the TPJ showed sensitivity to shared environmental effects when participants in this study were 7–9-years old (all prior to the COVID-19 pandemic)^22^. Not many studies examined recovery effects on brain development after high impact environmental experiences, but one prior study observed that the female brain shows immediate volume changes during pregnancy followed by recovery of some brain regions (e.g., hippocampus) one year later^88^. The present study suggests that the first months of the pandemic may have had largest effects on the developing brain, the TPJ specifically, but this effect became less pronounced in participants who were scanned later in the pandemic. Thus, this association may suggest that some brain regions become more resilient to adverse experiences of the COVID-19 pandemic. We added age at time point 1 to the analyses, since participants differed in TPJ cortical thickness prior to the pandemic with high cortical thinning in the *before* pandemic group on the first time point. All effects remained significant so it is unlikely that these effects are explained by developmental differences.

This study had several strengths, including a longitudinal design where the COVID-19 pandemic was included as a natural intervention. Furthermore, we used an age-matched control group and sufficient sample sizes. However, the study also has several limitations that should be addressed in future research. First, given the unexpected aspect of the pandemic, the study design was not pre-registered and should therefore be interpreted as an exploratory study. Effect sizes were relatively small and not all *p*-values survived correction for multiple comparisons. Therefore, future studies with larger sample sizes should replicate and confirm the results using a follow-up time point to examine the resilience or accumulating influences of the COVID-19 related pandemic effects. Second, the participants in this study were twins. We controlled for dependency in the data but given that participants grew up in the same families not all social experiences can be generalized to participants without twin-siblings. Third, even though the brain findings show compelling directions of COVID-19 pandemic effects on child development, in the current study these effects were not linked to behavioral findings. We limited our analyses to parent-reported empathy and prosocial behavior, which did not show any effects of the pandemic. Although the correlation between parent and youth-report measures of empathy and prosocial behavior showed to be high, future studies that additionally include self-report measures are recommended. Moreover, prior studies on child and adolescents’ behavior showed pronounced effects of the pandemic on multiple domains such as mood and social behavior^3,50,51,76,89^. Therefore, future studies could link neural development to other behaviors, including mood^39^.

Taken together, this study showed that experiencing the COVID-19 pandemic measures had accelerating effects on mPFC and hippocampal development in 9–13-year-old adolescents. These findings are partly in line with a prior cross-sectional study in a slightly older sample of 16-year-olds^39^. Moreover, TPJ maturation showed immediate effects followed by possibly subsequent recovery effects that returned to a normative pattern. This unique longitudinal study that includes a control group that was assessed before the pandemic, shows pandemic related effects on brain developmental patterns. The effects were subtle and should be confirmed in future longitudinal research in different age samples, including a focus on day-to-day social experiences during stressful events.

## Data Availability

Code that support the findings of this study is available on DataverseNL at https://doi.org/10.34894/0YUM34, data will be available upon request.
